# Plasmid stability analysis based on a new theoretical model employing stochastic simulations

**DOI:** 10.1371/journal.pone.0183512

**Published:** 2017-08-28

**Authors:** Olesia Werbowy, Sławomir Werbowy, Tadeusz Kaczorowski

**Affiliations:** 1 Laboratory of Extremophiles Biology, Department of Microbiology, Faculty of Biology, University of Gdańsk, Wita Stwosza 59, Gdansk, Poland; 2 Institute of Experimental Physics, Faculty of Mathematics, Physics and Informatics, University of Gdańsk, ul. Wita Stwosza 57, Gdansk, Poland; Institut National de la Recherche Agronomique, FRANCE

## Abstract

Here, we present a simple theoretical model to study plasmid stability, based on one input parameter which is the copy number of plasmids present in a host cell. The Monte Carlo approach was used to analyze random fluctuations affecting plasmid replication and segregation leading to gradual reduction in the plasmid population within the host cell. This model was employed to investigate maintenance of pEC156 derivatives, a high-copy number ColE1-type *Escherichia coli* plasmid that carries an EcoVIII restriction-modification system. Plasmid stability was examined in selected *Escherichia coli* strains (MG1655, wild-type; MG1655 *pcnB*, and hyper-recombinogenic JC8679 *sbcA*). We have compared the experimental data concerning plasmid maintenance with the simulations and found that the theoretical stability patterns exhibited an excellent agreement with those empirically tested. In our simulations, we have investigated the influence of replication fails (α parameter) and uneven partition as a consequence of multimer resolution fails (δ parameter), and the post-segregation killing factor (β parameter). All of these factors act at the same time and affect plasmid inheritance at different levels. In case of pEC156-derivatives we concluded that multimerization is a major determinant of plasmid stability. Our data indicate that even small changes in the fidelity of segregation can have serious effects on plasmid stability. Use of the proposed mathematical model can provide a valuable description of plasmid maintenance, as well as enable prediction of the probability of the plasmid loss.

## Introduction

Plasmids are self-replicating genetic elements that are separated from the host chromosome content [1]. They can be found in most bacteria, including pathogens, but also in archea [2] and yeast [3]. Some of them have a capacity to transfer themselves to a new host by conjugation or mobilization [4, 5]. However, being accessory non-essential pieces of DNA they may carry genetic determinants which under particular environmental conditions can provide a selective advantage to the host, including adaptation to specific habitats, production of virulence factors, resistance to antibiotics, heavy metals, degradation of xenobiotics or competition with other microbes [6–9]. Among many features that are characteristic to plasmids lifestyle [10], two are the most distinctive: (i) autonomous, self-controlled replication driven by cell proteins, and (ii) persistence even without selective pressure and at an obvious metabolic burden to the host [11–14]. The latter is especially challenging to analyze, as apart from general mechanisms that are involved in plasmid maintenance such as (i) random partition [15], (ii) active partition [16]; and (iii) plasmid addiction systems [17–19], there are other factors that play a pivotal role here. These include: horizontal gene transfer; positive selection for plasmid encoded genes, and compensatory adaptation [20, 21]. All of these make plasmid persistence a complex phenomenon that needs to be approached from different angles.

Many theoretical models were developed to study plasmid stability. One of them was based on the assumption that an appearance of plasmid-free cells could be a result of segregational instability of a resident plasmid [22]. This concept was further expanded by adding other parameters, which considered effects of post-segregational killing (PSK) of plasmid-free cells or different doubling time of cells with and without plasmids [23]. In another model, the authors implemented parameters concerning kinetics of DNA synthesis and plasmid segregation [24]. In turn, Nordström and Aagaard-Hansen, 1984 [25] presented a model in which they assumed that the plasmid copy number (PCN) parameter is determined only by two independent processes, replication and segregation of plasmid copies to daughter cells. According to this model, the plasmid stability is determined by the probability of formation of plasmid-free segregants. For plasmids segregated randomly (e.g. ColE1-type plasmids) this probability is given by *P*_0_ = 2^(1-n)^, where n = plasmid copy number in the mother cell [26]. The aforementioned models can be expanded further by assumption that each cell in a population does not bear the same number of plasmid copies, but rather, the plasmid content shows a Poissonian spread. This is connected with apparent non-random distribution of plasmid copies within the cell [27–29]. Thus, by solving a set of equations and comparing them with experimental results, quantified values of free parameters appearing in the equations can be obtained [30]. Another interesting model predicting dynamics of plasmid persistence in the absence of selection was described by Ponciano et al., 2007 [31]. This segregation and selection model (SS) considers growth dynamics of two bacterial populations, consisting of plasmid containing and plasmid-free cells, as a simple system of equations where it is assumed that at any generation, the abundance of the plasmid-free cells increases due to faults in plasmid segregation machinery. Most of mathematical models require many parameters that have to be taken into account. This brings some uncertainties to calculations. In fact, the greater the number of parameters a model uses the higher uncertainty is observed. Moreover, a large number of different parameters, in our opinion, complicates the synthetic comparison of plasmid maintenance in various settings.

As a model in our studies on plasmid maintenance we have used a naturally occurring plasmid pEC156 of *Escherichia coli* E158568 [32] that is a ColE1-type replicon [33]. This plasmid contains a toxin-antitoxin module consisting of genes coding for EcoVIII, a type II restriction-modification (RM) system comprising of a restriction endonuclease (R) and a DNA methyltransferase (M) that recognize the 5’-AAGCTT-3’ target sequence [34]. Another important feature of pEC156 revealed by computational analysis is the presence of a locus with similarity to the *cer* site of plasmid ColE1. This *cis*-acting site is involved in monomerization of plasmid multimers that arise by homologous recombination (RecF pathway) and thus increases the chance that each daughter cell will receive at least one copy of the plasmid upon division of the mother cell [26]. On the other hand, multimerization reduces the number of independently segregating plasmid units and thus seriously affects plasmid stability [26, 35]. Basically, plasmid multimers replicate more frequently than monomers. This results in their accumulation in the progeny of cells in which they initially arisen [36]. pEC156, like other ColE1-type high-copy plasmids, is segregated randomly as it does not carry any system that is required for an active partition. Our previous work had shown that both, the EcoVIII RM system and the *cer* site, are crucial for stable maintenance of pEC156 not only in *E*. *coli* but also in other enterobacteria [35, 37].

In the present study we propose a simple theoretical model to analyze a high-copy plasmid’s maintenance, with the smallest number of free parameters that could be fitted into the experiment. In this model, we have made the following assumptions: (i) at the start of the experiment, all of the cells contain the plasmid, (ii) upon cell division, the plasmids are segregated randomly between the two daughter cells, (iii) after cell division, the inherited plasmid molecules replicate only once per cell cycle, (iv) upon cell division, each daughter cell inherits a certain amount of plasmid molecules from the mother cell. The aforementioned assumptions were necessary to assess the probability of appearance of plasmid free cells. To theoretically analyze the plasmid stability, we have used the Monte Carlo method which represents a universal stochastic approach that is set to solve complex analytical problems. It relies on a very large number of similar random computation experiments performed and averaged to give the final solution. Each experiment runs with a defined number of input parameters and according to the basic set of rules reflecting the problem. The use of the proposed mathematical model can provide a valuable description of plasmid maintenance, as well as enable prediction of the probability of plasmid loss.

## Materials and methods

### Strains and plasmids

The following strains of *Escherichia coli* were used in this work: MG1655 (wild type (wt); [38]), MG1655 *pcnB*80 *zad*::Tn*10* [39]; and JC8679 *sbcA23*(*Rac*) *recB21 recC22* (hyper-recombinogenic; [40]). All bacteria were cultivated in Luria broth (LB) or Luria agar (LA) medium [41] at 37°C. When necessary, appropriate antibiotics were used at the following concentrations: chloramphenicol (Cm) 30 μg/ml and kanamycin (Km) 50 μg/ml. Plasmid pIB8 (EcoVIII R^+^M^+^*cer*^+^ Cm^R^, 5.2-kb) [42] was obtained from Dr. Iwona Mruk (University of Gdansk, Poland). Plasmid pRB1 (EcoVIII R^+^M^+^*cer*^−^ Cm^R^, 4.6 kb) and pRB2 (EcoVIII R^−^M^−^*cer*^−^ Cm^R^, 2.8 kb) were constructed in our laboratory and described in our previous report [35]. All aforementioned plasmids are available on request from the Collection of Plasmids and Microorganisms, University of Gdansk, Gdansk, Poland.

### Plasmid stability testing

Some of the experimental data concerning pEC156-derivatives’ stability was presented previously [35]. For the purposes of the present work, we analyzed stability of pRB1 and pRB2 in *E*. *coli* MG1655 and MG1655 *pcnB*. Some experiments were repeated, especially when bacteria were grown for 600 generations. They were performed essentially as described previously [35]. Plasmid stability data set is available as supporting information (S1 Appendix).

### Determination of plasmid copy number

The PCN of pEC156 derivatives was determined by droplet digital PCR (ddPCR) that enables absolute quantification of target DNA [43]. For this purpose, the *E*. *coli* strain carrying a pEC156 derivative was cultured in LB medium supplemented with an appropriate antibiotic to reach stationary phase, which become the start point (time zero) of the stability test or marked as generation “0”. Bacteria from 1 ml culture samples were harvested by centrifugation (5 min. 5000 × g) followed by total DNA isolation using the QIAamp DNA Mini Kit (QIAGEN). The concentration of DNA obtained was measured using NanoDrop 1000 UV-VIS spectrophotometer (NanoDrop Technologies, Wilmington).

To determine the plasmid copy number, two set of primers were used to amplify two single copy genes coding for D-1-deoxyxylulose 5’-phosphate synthase (*dxs*; reference gene) and chloramphenicol acetyltransferase (*cat*, target gene), respectively. The first primer set targeted the *dxs* gene located in the *E*. *coli* chromosome (F1: 5’-CGAGAAACTGGCGA-TCCTTA-3’; R1: 5’-CTTCATCAAGCGGTTTCACA-3’), while the second was for the *cat* gene carried by pEC156-derivatives ((F2: 5-TAAGA-GGTTCCAACTT-TCAC-3’; R2: 5’-CATTTTAGCTTCCTTAGC TC-3’). The use of primer sets for *dxs* and *cat* resulted in amplification of DNA fragments that were 113-bp and 95-bp in length, respectively. Standard ddPCR mixture (20-μl volume) contained 2× EvaGreen ddPCR Supermix (Bio-Rad) and appropriate primers at a concentration of 0.2 μM. After reaching the equilibrium (3 min, room temperature), the reaction mixture was dispensed into a droplet generator DG8 cartridge (Bio-Rad). Each oil compartment of the cartridge was filled with 70 μl of the droplet generation oil for EvaGreen (Bio-Rad), and approximately 20,000 droplets were generated at each well with the use of the QX200 droplet generator (Bio-Rad). Then, the emulsion (40 μl) was loaded onto a 96-well PCR plate (Eppendorf). The plate was then heat sealed using the PX1 PCR Plate Sealer (Bio-Rad), and placed in a Mastercycler ep gradient S thermocycler (Eppendorf). The following thermal cycling settings were used: 95°C for 5 min; 35 cycles of 95°C for 30 s, 58°C for 30 s, 72°C for 1 min; and a final step at 72°C for 1 min. After amplification, the plate was transferred into a QX200 Droplet Reader (Bio-Rad), where the droplets from each reaction were annotated as positive or negative, based on their fluorescence amplitude. The number of positive and negative droplets in each channel was used to calculate the concentration of the target (*cat*) and reference (*dxs*) DNA sequences. The plasmid copy number was calculated by dividing the concentration of *cat* (copies/μl) by *dxs* (copies/μl).

### Determination of multimer sizes

The hyper-recombinogenic strain of *E*. *coli* JC8679 *sbcA* was used as host for pEC156-derivatives (pRB1 and pRB2). Plasmids were isolated using the Plasmid Mini AX kit (A&A Biotechnology) which is based on the alkaline lysis method [44]. This results in isolation of plasmid units mainly in the form of covalently closed circular molecules. Position of plasmid monomers and dimers was determined by partial digestion with restriction enzymes that cut the analyzed plasmids at a unique site, as described by others [45]. It was assumed that plasmid multimers isolated from the hyper-recombinogenic strain JC8679 and separated in 0.8% agarose gel represent supercoiled DNA molecules in the form of monomers, dimers, trimers, etc. (x1, x2, x3, etc.; S1 Fig, panel A and C). In the next step, we have densitometrically determined the mobility of each plasmid multimeric form (S1 Fig, panel B and D). These consecutive multimeric forms were clearly distinguished from one another and their peaks plotted against the theoretical size of the multimer comprised a calibration curve (S1 Fig, panel E).

### Theoretical model

In order to develop a theoretical model that predicts fluctuations of the plasmid copy number within population of cells during a typical growth, the following assumptions were made: (i) plasmid molecules replicate only once per cell cycle, (ii) upon cell division, each of the two daughter cells inherits a certain amount of plasmid units from the mother cell.

#### Model setup

The simulation runs from the point “0”, in which we assume that the population consists of plasmid containing cells as a starter culture and is cultivated in a medium supplemented with an antibiotic that exerts a selective pressure. It was suggested by others [46–49] that such populations can be described by a Gaussian function with two parameters: (i) average number of plasmid units per cell (*N*_0_) and (ii) a standard deviation (σ) which measures how the numbers are spread out from an average value of plasmid units per individual cell:

$$M(n(0))=M_{0}\frac{1}{\sigma \sqrt{2\pi }}\exp\left(-\frac{1}{2}{\left(\frac{n-N_{0}}{\sigma }\right)}^{2}\right),$$ (1)

where exp(x) means the exponential function e^x^, and *M*_0_ is the overall initial number of cells.

In Eq 1, *M*(*n*(0)) defines the number of cells at “0” generation (time zero) which contain “n” units of the plasmid. Gaussian distribution in statistics is considered as an approximation of a more fundamental Poisson distribution. Therefore, in simulations, we have assumed that the initial population of cells is described by the Poisson distribution. There are differences between Poisson and Gaussian distribution: (i) the first is asymmetric, especially at small values of *N*_0_, (ii) and it is described by only one parameter, the mean plasmid copy number *N*_0_ (also known as PCN). In the Gaussian distribution, the standard deviation σ (the spread of the plasmid copy number per cell) is an independent parameter. In the Poisson distribution, the standard deviation is directly related to the mean value of PCN per cell *N*_0_ and the spread of the results is equal to $\sqrt{N_{0}}$. However, for *N*_0_ values greater than 10 both functions are almost identical under condition that σ^2^ = *N*_0_.

$$M(n(0))=M_{0}\frac{N_{0}^{n}}{n!}\exp(-N_{0}),\;M(0(0))=0.$$ (2)

The condition *M*(0(0)) = 0 takes into account that the starter culture was cultivated under antibiotic pressure, which imposes that cells which fail to inherit the plasmid would die. At this point, we have assumed that the cell needs at least one copy of plasmid to resist the action of an antibiotic.

Nevertheless, we have applied in our model the Poisson distribution to the initial population of cells. In a situation where there are no special conditions, variations in the number of plasmid units per cell associated with the random fluctuations could relate to the average PCN of the population. In case of RK2, it was shown that distribution of plasmid clusters is asymmetric, tending to lower the values of plasmid foci per cell and the spread out of the results is correlated with the mean value, reflecting the Poisson distribution [27]. This *in situ* observation led us to choose the Poisson distribution as an initial distribution. The aforementioned description refers to the preparatory phase of the experiment in which the plasmid bearing cells were cultivated in the presence of an antibiotic. We assume that at this point all cells contained the plasmid. Thus, the initial population is represented by incipient distribution (Eq 2), depended only on the plasmid copy number *N*_0_(g = 0), which can be determined experimentally and was used as the input parameter in the proposed theoretical model.

#### Bacterial cell cycle—plasmid replication

In a growing cell, the plasmid content doubles during each cell cycle. Upon cell division, plasmid units that have been replicated are segregated into two daughter cells. The process of DNA replication is a complex one, and is controlled by many factors. This complexity is well illustrated by the existence of precise regulatory circuits that keep plasmid copy number at a certain level, thus plasmid content is secured from reaching the extremes (too many or too few plasmid units per cell). This can be overcome by introducing a function that is depended on the current number of plasmid units in the host cell, which will reduce the replication multiplier when the number of plasmid molecules reaches the maximal capacity of the cell (*n*_max_). This is given by:

$$R(n)=1+\frac{a}{1+b^{\left(n-n_{\max}\right)}},$$ (3)

where *a* and *b* are some empirical parameters describing the dependency of the replication multiplier function on the current number of plasmid units.

In our model, we have assumed that during each cell cycle the parent cell that contains *n*(*g*-1) plasmid units (g = the number of generations), increases the number of plasmid units which are segregated upon cell division into two daughter cells, as given by:

$$n(g)=R(n)\cdot n(g-1)-n_{\alpha },$$ (4)

where *n*_α_ is the random number of replication errors that result in replication-defective plasmid units. Because we have no knowledge of the exact values of *a*, *b* and *n*_max_ parameters from Eq 3, in order to make quantitative comparison between different plasmid-bacteria systems possible, in Eq 4 we have made an assumption that plasmid content is doubling during each cell cycle *R*(*n*) = 2.

The random number of plasmid replication fails, *n*_α_ from Eq 4, is subjected to stochastic processes and is determined at each step of a simulation. In order to determine *n*_α_ we use the exponential function:

$$P(n_{\alpha })=e^{-n_{\alpha }/\alpha },\;n_{\alpha }=[-\alpha \ln P],$$ (5)

where α is the parameter describing the probability distribution *P*(n_α_). At this step of simulation, a random number generator draws a *P* number (in the range 0–1) and, depending on its value sets, the number of *n*_α_. Thus, the final numbers of plasmid units in two daughter cells are determined including stochastic variations in the processes of plasmid DNA replication.

#### Bacterial cell cycle—cell division

At the stage of cell division, the plasmid content reaches its maximum level. Plasmid units are segregated randomly between the two daughter cells and the number of plasmid units in each new cell is given by:

$$n_{1}(g)=[\delta \cdot n(g)],\;n_{2}(g)=n(g)-[\delta \cdot n(g)],$$ (6)

The δ parameter takes into account the uneven segregation of plasmid units into daughter cells, and is ranging from 0 to 0.5. The value of δ = 0.5 means that at the cell division, both daughter cells will receive the same number of plasmid units. In the case of smaller values of δ, one daughter cell will receive more plasmid molecules than the other one. The δ parameter can be interpreted in two ways. Firstly, it is associated with partition fails, thus δ is an important parameter for plasmid maintenance not only in the case of partition by an active process, but also by random segregation. All low-copy number plasmids encode at least one partition system [50], which suggests that this parameter would be very important and useful in the case of large plasmids. The second interpretation of the δ parameter is connected with high-copy number plasmids that are segregated randomly. In this case, the partition fails are related to faults in the resolution of plasmid multimers that are the effect of a recombination-dependent process.

In the next step, the number of cells *M*(*n*(*g*)) that contain *n*(*g*) plasmid units at *g* generation is calculated:

$$M(n_{1}(g))=M(n(g-1))+M'(n_{1}(g)),$$

$$M(n_{2}(g))=M(n(g-1))+M'(n_{2}(g)),$$ (7)

where *M*’(n_1,2_(*g*)) are the number of cells that have already existed in the population with the *n*_1_ and *n*_2_ plasmid units.

The method used for calculation of the number of cells in a given population is outlined in S2 Fig. There are three cells (generation *g*-1) bearing the following numbers of plasmid units: one with 2, another with 1 and the third with no plasmid. The arrows indicate changes in segregation of the plasmid content. For each cell, the number of plasmid units per each daughter cell is determined and on this basis the number of cells that contain *n*(*g*) plasmid units is calculated. In the example presented in S2 Fig we see that one cell without any plasmids after division gives two plasmid-free cells *M*(0(*g*)) = 2. Cells with one plasmid after division split into two cells each having one plasmid unit (this means that in the replication phase the plasmid content doubled and the resulting two plasmid units were segregated evenly between the two daughter cells). However, in case of the cell with two plasmid units we see that due to some random fluctuations at the stage of DNA synthesis, their replication resulted only in one additional unit completing the cell cycle with three plasmid units. During cell division, one daughter cell received two plasmid units while the other cell inherited only one, increasing the size of population of bacteria with one plasmid unit per cell.

#### Bacterial cell cycle—post-segregational cell killing

The loss of a plasmid that bears a toxin-antitoxin module results in the formation of plasmid-free cells that might be eliminated from the population by post-segregational cell killing. The number of such plasmid-free cells is given by:

M(0(g))=[β⋅M(0(g))], (8)

where the β parameter sets the level of death rate of plasmid-free cells due to post-segregational cell killing (β<1). Because the number of plasmid units in a cell is a natural number, the brackets [] in Eqs 5, 6 and 8 mean that in simulations we take the whole number without a fractional component from the results obtained.

When the initial distribution is somehow modified by special experimental settings before the “0” generation (time zero), this would have negligible influence on the results of the simulation. For example, if an initial distribution would be in the form:

$$M(n(0))=\left\{ \begin{array}{ll} M_{0}, & \;n=N_{0}\\ 0, & n\neq N_{0} \end{array}\right.,$$ (9)

where *M*_0_ is the total initial number of cells that have *N*_0_ plasmids per cell (this corresponds to the situation where due to antibiotic pressure all cells bear at least one plasmid unit). In such a case, as is shown in S3 Fig, in the analyzed population we will observe the appearance of random fluctuations in the distribution of plasmid units after a few generations.

#### Measurable quantities derived from the theoretical model

The schematic overview of the plasmid stability model is shown in S4 Fig (panel A). The simulation starts with one cell carrying four plasmid units. During the first three generations we can see how stochastic processes lead to the appearance of plasmid-free cells. Red crossed circles indicate the appearance of replication-defective plasmid units with the rate determined by the α parameter. S4 Fig (panel B) shows the changes in plasmid copy number presented as percentage of the analyzed population. Arrows indicate values of the PCN. S4 Fig (panel C) shows changes in the plasmid stability and S4 Fig (panel D) shows plasmid copy number as a function of time (generation number). As a result of simulations, for each generation we are able to obtain the distribution of cells with different plasmid copy number in a given population. From these distributions, it is possible to directly obtain the following observables (see S4 Fig, panel C and D):

Distribution of the percentage of cells with a given number of plasmid units:

$$P(n,g)=\frac{M(n(g))}{\sum_{n=0}M(n(g))},$$ (10)

Plasmid stability:

$$S(g)=\frac{\sum_{n=1}M(n(g))}{\sum_{n=0}M(n(g))},$$ (11)

The average number of plasmid units:

$$PCN(g)=\frac{\sum_{n=0}n\cdot M(n(g))}{\sum_{n=0}M(n(g))},$$ (12)

In principle, all observables (Eqs 10–12) can be studied in the laboratory using experimental approaches.

#### Effect of replication errors

In this section, we analyze the influence of the α and δ parameters on plasmid stability. The first parameter (α) takes into account a random appearance of the replication errors resulting in replication-defective plasmid units. S5 Fig shows an average of 50 simulations calculated for different values of the α parameter in the range of 0.5–3.0. The probability distribution function for different values of the α parameter is shown in S6 Fig. The higher the α parameter is the more probable that the cell will lose a certain amount of plasmid units. In this simulation, the other parameters were fixed (β = 0, and δ = 0.5). The initial plasmid distribution was taken from Eq 9 with *N*_0_ = 20 and 5. Thus, we have assumed that the only reason why plasmid-free cells have appeared is due to fluctuations in the replication process resulting in replication-defective plasmid units. In consequence, at cell division, the number of plasmid units available for segregation is lower than 2*n*. This may result in daughter cells inheriting less plasmid units when compared to the mother cell at the same phase of the cell cycle. The described behavior after several rounds of replication would lead to the formation of plasmid-free cells. As shown in S5 Fig (panel A and B), an increase in the replication errors results in an early emergence of plasmid-free cells. Another conclusion is that at a given probability of replication errors, the plasmid stability is dependent only on the initial plasmid copy number (*N*_0_). The 3-D plots in the S5 Fig (panel C and D) show the behavior of the relative distributions of the percentage of populations for *N*_0_ = 20, and two values of the α parameter: 0.5 (S5 Fig, panel C) and 1.5 (S5 Fig, panel D). With each next generation, the PCN shifts toward the smaller values of a single cell copy number. This will end in plasmid-free cells. These results are in agreement with the *in situ* observations [27], where the shift in cellular distribution of plasmid units in the exponential growth phase and 1.5 hours later was observed.

#### Effect of plasmid partition errors

The second possible cause that could lead to the appearance of plasmid-free cells is associated with plasmid segregation, related to the δ parameter. Partition mechanisms could lead to the uneven segregation of plasmid progeny to daughter cells, resulting in the loss of plasmid content after a few generations. S7 Fig (panel A and B) shows the fraction of plasmid containing cells estimated for different values of the δ parameter, in the range of 0.3–0.49. In these simulations we have used the initial plasmid distribution described by Eq 9 for *N*_0_ = 20 and 5; (α, β = 0). The 3-D plots in S7 Fig (panel C and D) show the behavior of the relative distributions of the percentage of populations for *N*_0_ = 20, and two values of the δparameter: 0.49 (S7 Fig, panel C) and 0.40 (S6 Fig, panel D). We observe that in case of uneven partitioning (δ<0.5), plasmid distribution is not similar to that depicted in S5 Fig where an effect of the α parameter was analyzed. Our simulation suggests that after several generations we could observe appearance of plasmid-free bacteria and a population of bacteria characterized by diverse PCN (S7 Fig, panel C and D).

#### Statistical model code for analysis of plasmid stability

The code used to run the statistical model is freely available to all users (S2 Appendix).

## Results

### Application of the theoretical model to analysis of the pEC156-derivatives’ maintenance

The theoretical model developed in this study was applied to the analysis of maintenance of pEC156-derivatives: pIB8 (EcoVIII R^+^M^+^*cer*^+^), pRB1 (EcoVIII R^+^M^+^*cer*^−^) and pRB2 (EcoVIII R^−^M^−^*cer*^−^). The general overview of the model is given in the Materials and methods section. The initial parameters are the Poisson distribution with *N*_0_ values adopted from PCN measurements, *N*_0_ values together with the doubling time of bacteria are given in Table 1. In the simulation, for each plasmid-bacteria pair, we have tried to independently establish the values of the α and δ parameters (Table 2), for which there is a satisfactory agreement with the experimental results presented in our previous report [35].

**Table 1:** Average number of plasmid units per cell (*N*_0_) in starter cultures cultivated under antibiotic pressure. Doubling time for each bacterial strain was determined using standard titer procedure.

Plasmid								
		None	pIB8 (R^+^M^+^*cer*^+^)		pRB1 (R^+^M^+^*cer*^−^)		pRB2 (R^−^M^−^*cer*^−^)	
		Doubling time (min.)	PCN (*N*_0_)	Doubling time (min.)	PCN (*N*_0_)	Doubling time (min.)	PCN (*N*_0_)	Doubling time (min.)
*E*. *coli* strain	MG1655 (wt)	21.3±2.4	14±2	19.5±4.0	16±3	20.8±2.4	15±3	19.1±2.6
	MG1655*pcnB*	42.8±4.2	1.8±0.2	45.2±3.4	1.4±0.4	47.4±6.0	1.6±0.5	44.7±3.3
	JC8679 *sbcA*	22.6±3.4	21±3.4	22.0±3.1	26.3±0.5	20.5±2.2	29.5±2.7	21.4±2.5

**Table 2:** Experimentally derived values of α, β, and δ parameter.

		Effect of replication fails (α)			Effect of multimer resolution fails (δ)		
Plasmid							
		pIB8 (R^+^M^+^*cer*^*+*^)	pRB1 (R^+^M^+^*cer*^-^)	pRB2 (R^-^M^-^*cer*^-^)	pIB8 (R^+^M^+^*cer*^*+*^)	pRB1 (R^+^M^+^*cer*^-^)	pRB2 (R^-^M^-^*cer*^-^)
*E*. *coli* strain	MG1655 (wt)	0.25^(a)^ (α)	0.29 (α)	1.20 (α)	0.49±0.01 (δ)	0.49±0.01 (δ)	0.40±0.02 (δ)
		100% (β)^(b)^	100% (β)	80%(β)	95% (β)	97% (β)	100% (β)
	MG1655 *pcnB*	0.51 (α)	0.64 (α)	0.52 (α)	0.45±0.05 (δ)	0.38±0.05 (δ)	0.45±0.05 (δ)
		100% (β)	100% (β)	80% (β)	99% (β)	100% (β)	100% (β)
	JC8679 *sbcA*	0.35 (α)	0.80 (α)	0.94 (α)	0.49±0.01 (δ)	0.39±0.01 (δ)	0.37±0.02 (δ)
		100% (β)	100% (β)	80% (β)	98% (β)	100% (β)	100% (β)

^(a)^ For α parameter we estimate relative uncertainties to be 15% of a given value.

^(b)^ β-post-segregational killing factor.

#### Analysis of the pIB8 maintenance

**Fig 1:**
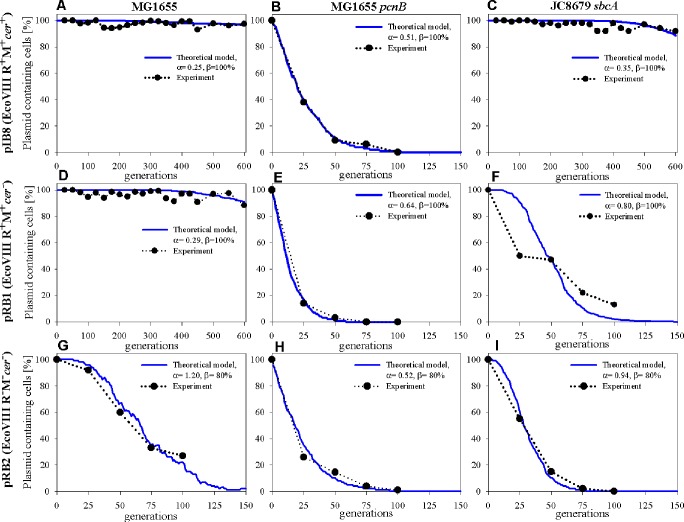
Comparison of experimental data obtained for pEC156-derivatives (pIB8, pRB1 and pRB2) maintenance in *E*. *coli* MG1655, MG1655 *pcnB*, and JC8679 *sbcA* with the theoretical model. Experimental points in panel B, E and H were taken from our previous report [35]. Continuous blue lines are best fit simulations for a given α parameter.

Plasmid pIB8 is a derivative of pEC156 possessing all fully functional elements responsible for its maintenance (phenotype: EcoVIII R^+^M^+^*cer*^+^). Comparison of the experimental data for pIB8 maintenance in *E*. *coli* MG1655, MG1655 *pcnB*, and JC8679 with the theoretical model is shown in Fig 1 (panels A, B and C). Experimental results indicate that pIB8 is stably maintained not only in MG1655 but also in a hyper-recombinogenic JC8679. The PCN values for this plasmid are 14±2 and 21±3.4, for MG1655 and JC8679, respectively. A reduced stability of pIB8 was observed with MG1655 *pcn*B due to the low plasmid copy number in this particular strain (PCN = 1.8±0.2). It is known than in *E*. *coli* strains carrying dysfunctional *pcnB* gene, the antisense RNA molecule (RNA I) involved in controlling replication of ColE1-type plasmids is more stable than in the wild-type bacteria, affecting the plasmid copy number [51]. Based on the simulation, we have found that the observed pIB8 stability pattern in the *E*. *coli* strains tested, the best fit for the α parameter is 0.25 and 0.51 for MG1655 and MG1655 *pcn*B, respectively (Table 2). This indicates that according to Eq 5 and for α = 0.51 there is an 86% chance that all plasmid units will replicate correctly (*n*_α_ = 0), 13% chance that one plasmid unit would fail to replicate (*n*_α_ = 1) and 1% that two plasmid units would fail (n_α_ = 2). For α = 0.25, this would correspond to 99% chance that all plasmid units will replicate correctly (*n*_α_ = 0), and 1% chance that one plasmid unit would fail to replicate (*n*_α_ = 1). The values of the α parameter differ for both strains, and the only reason for the observed difference in the stability pattern is the pIB8 plasmid copy number in cells at the 0 generation point (time zero), which was determined to be 14±2 and 1.8±0.2 for MG1655 and MG1655 *pcn*B, respectively (Table 1). In case of the hyper-recombinogenic JC8679 strain (α = 0.35; Table 2), we observed a stability pattern similar to MG1655 (Fig 1C and S8 Fig, panel C), as both strains are recombination–proficient (*rec*^*+*^ phenotype).

**Fig 2:**
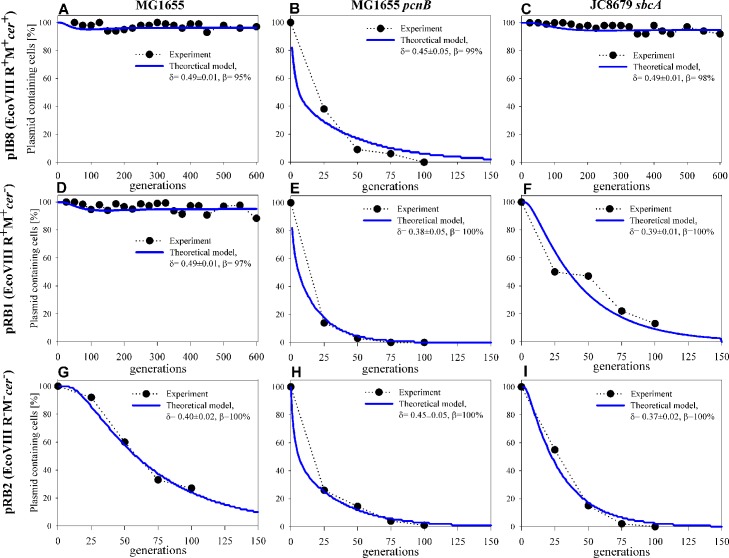
Comparison of experimental data obtained for pEC156-derivatives (pIB8, pRB1 and pRB2) maintenance in *E*. *coli* MG1655, MG1655 *pcnB*, and JC8679 *sbcA* with the theoretical model. Experimental points in panel B, E and H were taken from our previous report [35]. Thick lines are the best fit simulations for a given δ parameter.

The second possible cause of the decrease of pIB8 stability would be uneven partitioning of new plasmid molecules among two daughter cells. Fig 2 (panel A, B and C) shows the results of the theoretical analysis of pIB8 maintenance in the aforementioned *E*. *coli* strains, where the effect of the δ parameter associated with uneven distribution was tested. The distribution of cells with different plasmid copy numbers is shown in S9 Fig (panel A, B and C). Let us recall that δ = 0.5 means that plasmids are evenly distributed between the two daughter cells. We have found that in all strains tested, the δ parameters are only slightly smaller than 0.5, which means that plasmids are segregated by the parent cell almost evenly among descendants. Nevertheless, even small differences in distribution of plasmid progeny will affect the plasmid stability, leading to a rise in plasmid-free cells. This kind of uneven distribution can be a result of formation of plasmid multimers. In this case, the δ parameter would indicate the probability of such multimerization events. Thus, we should expect an increase in the plasmid copy number after several generations. This will result in appearance of cells with greater and greater number of plasmids per cell than the PCN characteristic for bacteria representing generation “0”. S10 Fig presents the calculated distributions of single cell plasmid copy numbers for plasmid pIB8 in the MG1655 strain (the same data as in the Fig 2A). At the beginning of the experiment, the mean PCN value was 14 plasmid units per cell (Table 1). Uneven segregation of plasmid units to descendent cells lead to changes in the mean plasmid copy number distribution, increasing it to 22 and 32 plasmid units per cell after 250 and 600 generations, respectively. Measurements of PCN for this system at 250 and 600 generations supports this notion. After 600 generations, the PCN has almost doubled when compared to the value determined for bacteria representing the generation “0”. The PCN determined from samples of the MG1655 strain with pIB8 plasmid for 0, 250 and 600 generation gave values of 11±2, 13±2 and 19±2, respectively.

#### Analysis of pRB1 maintenance

Plasmid pRB1 (EcoVIII R^+^M^+^*cer*^−^) is a derivative of pIB8 deficient in the *cer* site which is part of a multimer resolution system. In case of plasmid multimers, the value of PCN determined by dd-PCR does not correspond with the actual number of multimer units. In fact, the method used for PCN determination fails to distinguish between a plasmid being in a monomeric or multimeric forms. It only indicates the average number of plasmid units. In order to estimate the average number of multimers in the cells, the value of PCN determined by dd-PCR should be divided by the mean rank value of the multimers:

$$PCN=\frac{dd-PCR}{\rho },\;\rho =\sum_{k}\rho_{k}k,$$ (13)

where *k* is an order of multimer and dd-PCR is the value of the plasmid copy number as determined by the dd-PCR technique. The *ρ*_k_ is normalized distribution of the multimers, and can be obtained by electrophoretic separation of the supercoiled plasmid DNA.

**Fig 3:**
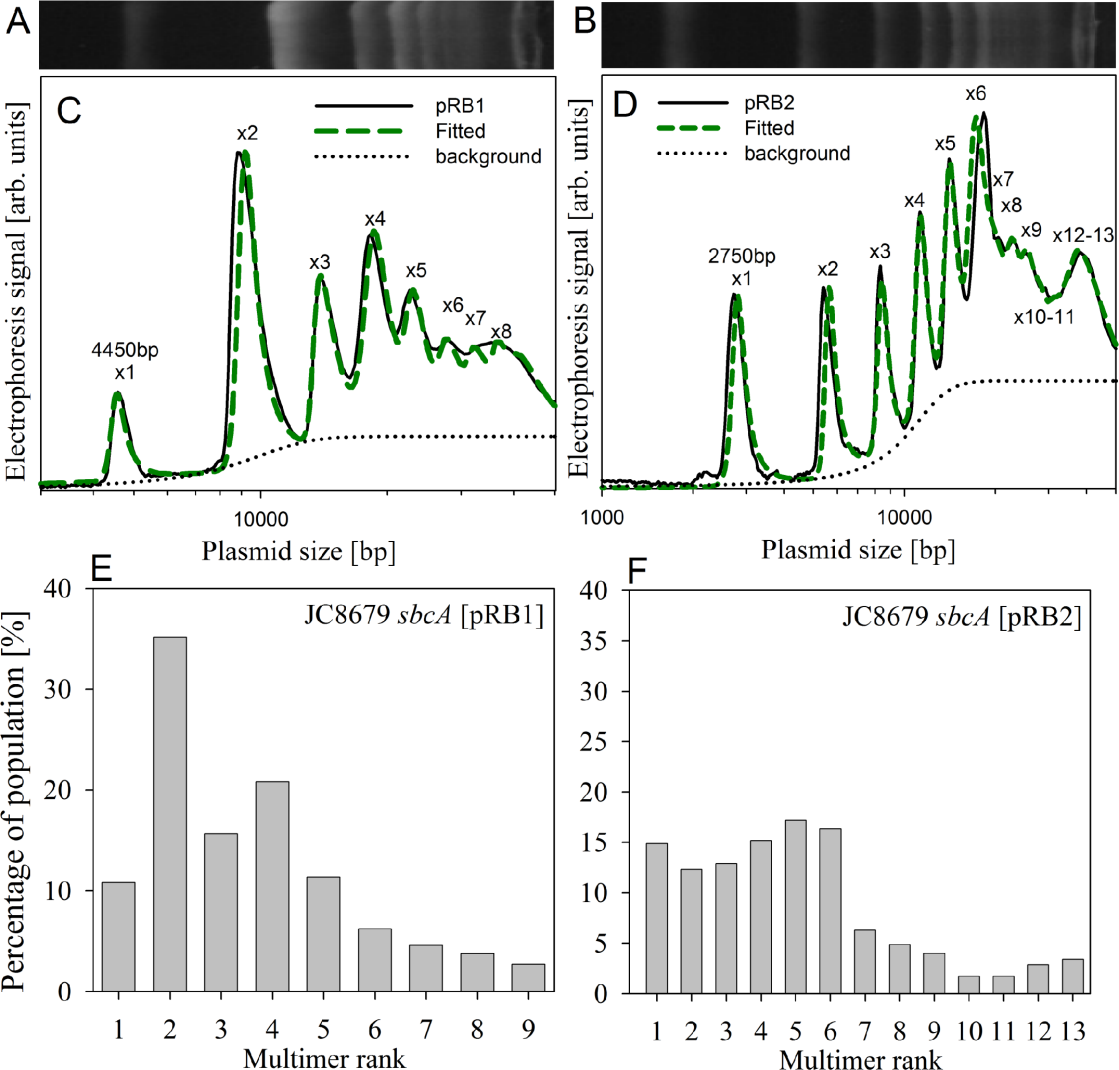
Densitometric evaluation of the multimer pattern of pRB1 and pRB2 isolated from hyper-recombinogenic *E*. *coli* JC8679 *sbcA* (generation “0”). Panels A and B show agarose gel electrophoresis of plasmid multimers for pRB1 and pRB2, respectively. Panels C and D show multimer forms determined by densitometry scanning. The dashed green line was calculated assuming that the observed pattern represents different types of plasmid multimers (×1- monomers, ×2- dimers, ×3- trimers, etc., as given by Eq 14). Panels E and F show distribution of pRB1 and pRB2 plasmid multimers.

Fig 3 shows agarose gel electrophoresis for pRB1 (panel A) and pRB2 (panel B) multimers isolated from a hyper-recombinogenic *E*. *coli* JC8679 strain. In order to analyze the observed plasmid pattern and find the fractions of different multimer sizes we have calculated the theoretical pattern as a sum of several peaks, each peak is described by the empirically matched formula:

$$I(x)=\frac{T}{1+\exp\left(-\left(\frac{x-x_{T}}{c}\right)\right)}+\sum_{k=1}\left(\frac{N_{k}}{1+{\left(\frac{x-k\cdot x_{0}}{\sigma_{k}/2}\right)}^{2}}\cdot \frac{1}{1+\exp\left(-\frac{\left(x-k\cdot x_{0}\right)}{b}\right)}\right),$$ (14)

where *N*_k_ is the relative abundance of a peak, *x* is the size of a DNA on the gel after linearization, *x*_0_ is the size of the single plasmid fragment (*k* = 1), *k* is the rank of the plasmid, *σ*_k_ is the width of the peak, *b* is the peak asymmetry parameter and *T*, *xT* and *c* are the parameters describing the background. Dashed lines in Fig 3 (panel C and D) show a plasmid multimer pattern derived from agarose gel electrophoresis. On the other hand, Fig 3 shows also distribution of pRB1 (panel E) and pRB2 (panel F) multimers isolated from the *E*. *coli* JC8679 strain. For each plasmid isolated from the hyper-recombinogenic JC8679 strain we have identified multimers in a range up to 10. The *N*_k_ values in Eq 14 can be used to determine the normalized distribution of multimers *ρ*_k_.

Plasmid RB1, despite the lack of a *cer* site, has the same number of plasmid copies at 0 generation as pIB8 (Table 1). Experimental results indicate that pRB1 is stably maintained only in MG1655 but not in a hyper-recombinogenic JC8679 and MG1655 *pcn*B. This instability is caused by plasmid multimerization and low copy number, respectively. When compared to pIB8, the obtained values for the α parameter for pRB1 stability testing in MG1655 and MG1655 *pcnB* are similar (Fig 1, panel D, E and F, Table 2). Assuming the hypothesis that appearance of plasmid free cells is related to the random replication fails, the α parameters of the best fit are higher by 20% and 14% than for the pIB8 plasmid. We have also found that the effect of the β parameter is negligible (β> = 99%) in the *E*. *coli* MG1655 and MG1655 *pcnB* strains.

The results shown in Fig 2 (panel D, E and F) for pRB1 support a notion that uneven partition of the new plasmid units affects the plasmid stability. Values of the δparameter derived from the theoretical model for pRB1 (Table 2) are slightly lower than those for pIB8 (see Fig 2 for comparison). This could be explained by inability to resolve the plasmid multimers, as pRB1 is devoid of the *cer* site (Fig 2, panel F; S9 Fig, panel F). The formation of plasmid multimers in *rec*^+^ strains is variable and in some cases reversible [36, 52]. In this case we didn’t have to use the βparameter to obtain good agreement with the experiment, indicating that uneven partitioning contributes to formation of the plasmid-free cells.

#### Analysis of pRB2 maintenance

Plasmid pRB2 (EcoVIII R^−^M^−^, *cer*^−^) is a derivative of pIB8 deprived of the R-M system, as well as the *cer* site. Its maintenance was tested in *E*. *coli* MG1655 (wt), MG1655 *pcn*B and JC8679 *sbcA*. The results obtained suggest that dual deficiency (EcoVIII R^−^M^−^, *cer*^−^) resulted in low stability of pRB2 in the strains tested due to plasmid multimerization and lack of EcoVIII RM system (Fig 1, panel G, H and I). By comparative analysis of results derived from simulation and experimental settings for MG1655 and MG1655 *pcnB*, we observe large differences between values of the αparameter in both strains (Table 2). The random fluctuations in replication fails are very high in case of MG1655, α = 1.20 which corresponds to 25% chance that one plasmid will fail to replicate, 11% chance that two will fail, 5% chance that three, and 2% chance that 4 plasmid will fail to replicate in each generation. The results obtained for JC8679 are similar to those observed for MG1655 (Fig 1, panel I). However, in the JC8679 strain, when assuming that cells are losing plasmids due to the replication fails, to obtain a good agreement with the experiment it was necessary to set β = 80% (this assumes that in each generation, 20% of cells without a plasmid will die). Results of simulations shown in Fig 2 (panel G, H and I) indicate that the decrease in plasmid-containing cells depends on the uneven partitioning of plasmids (δ parameter). Experimental results indicate that pRB2 is unstably maintained in all of the strains tested. The δ parameter values are smaller than those for the pIB8 and pRB1 plasmids. Based on the simulations we found that in each generation step, MG1655 or JC8679 mother cells segregate pRB2 plasmid to two daughter cells in proportion of 40:60 and 37:63, respectively.

## Discussion

Despite the significant metabolic burden placed on the host cells, naturally occurring plasmids are usually stably maintained. This kind of persistence is a result of an equilibrium between plasmid replication, multimer resolution and partition. A concerted action of all three processes is responsible for the rare appearance of plasmid-free cells [52]. It is not unusual for a single plasmid to possess several maintenance systems that ensure its stability [53–55].

In the study presented here, we have analyzed experimental data on the maintenance of the plasmid pEC156 derivatives with the help of a theoretical model, based on the Monte Carlo simulations. Our research model is applicable to high-copy plasmids with a ColE1-type replicon that was shown to be functional throughout the whole bacterial cell cycle [56]. These types of plasmids replicate through the theta type mechanism which has been studied extensively [57, 58]. The most crucial step involves synthesis of the RNA II transcript which forms a hybrid with the template that serves as a primer in plasmid replication after processing with RNase H. The process of primer formation is controlled by RNA I molecule that is complementary to the 5’ region of the RNA II transcript and serves as its antisense inhibitor. Binding of RNA I to the primer transcript cause conformational rearrangements in RNA II that negatively affect formation of a persistent hybrid with DNA template, and as a consequence blocking the initiation of DNA synthesis [59]. In general, newly synthesized plasmid units are thought to be segregated randomly upon cell division; however, recent studies challenged this notion. It was shown that not all plasmid units are evenly distributed throughout the host cell but a significant portion of them preferentially localize to the cell poles as distinctive multifocal clusters [27, 28, 60–62]. This resembles the behavior of a bacterial nucleoid prior to cell division, suggesting the existence of an unknown mechanism responsible for active segregation of plasmid molecules [29]. In addition, such uneven distribution and plasmid clustering reduces the number of units that are available for segregation to daughter cells and increases the probability of plasmid loss. However, numerous molecular mechanisms have evolved to stably maintain the plasmid content. In case of pEC156, these include: (i) a cis-acting *cer* site involved in resolution of plasmid multimers, (ii) the presence of EcoVIII RM system, and (iii) plasmid copy number control [35].

In the work presented here, we were especially interested in using a theoretical model to provide clues explaining the effect of how plasmid maintenance is affected by fluctuations in the distribution of plasmid units in the host cells. As such, we have investigated the effect of replication fails that produce defective plasmid molecules, as well as uneven segregation of plasmid units into daughter cells. In the proposed theoretical model, we have introduced the random fluctuations affecting plasmid replication and segregation. By parametrization of these events (see Table 2), we have compared the experimental data with the simulations and have determined the frequencies of these events for which theoretical stability patterns displayed an excellent agreement with those examined in the laboratory. In our simulations, we have investigated influence of replication fails (α parameter) and uneven partition as a consequence of multimer resolution fails (δparameter), and the post-segregation killing factor (β parameter). All of these factors act at the same time and affect plasmid inheritance to different extents. The only input parameter was the mean PCN at the beginning of the stability experiments, determined by droplet digital PCR method. Based on the developed theoretical model we can conclude that errors, either in plasmid replication or in plasmid segregation, will lead to appearance of plasmid-free cells. The obtained results indicate that among aforementioned errors the most serious effect is associated with plasmid segregation. Data on stability of the pEC156-derivatives deficient in the *cer* function suggest that multimerization is a key factor that affects even distribution of plasmid units between daughter cells at the cell division.

In the case of plasmids and strains that were used in the present work, it is difficult to explain the observed experimental stability pattern based only on differences of the doubling time of cells with and without plasmids, as was suggested by the early theoretical models [22, 31]. The experimentally determined doubling times of the plasmid-free and plasmid-bearing cells appear to be almost identical within the experimental error bars (see Table 1). In most models, it was assumed that a plasmid bearing cell could with certain probability become a plasmid-free cell, instantly losing all plasmid units it contains. In contrast to this approach, the model presented here assumes a gradual reduction of plasmid content that after several rounds ends with a plasmid-free cell. With a single parameter, it is more convenient to make a comparison between various plasmid-host systems. Our experimental stability curves show a behavior common for most of the known systems. Although other theoretical models can be satisfactorily fitted to the experimental data, they usually need more fitting parameters in comparison to the model presented in this report. These extra parameters themselves may constitute a separate issue introducing additional uncertainty and complexity to interpretation of the experimental results.

Plasmid stability is heavily dependent on the evenness of segregation of the newly replicated plasmid units. Uneven segregation of plasmid units to daughter cells can be explained by either: (i) random fluctuations in plasmid segregation to daughter cells as suggested by Lau et al., 2013 [63], or (ii) spontaneous formation of multimers and/or tangling of the independent plasmid molecules that are later segregated unevenly to the descendant cells [27, 61, 64]. All of those mechanisms could be interpreted as the δ parameter from Eq 4. Based on the simulations we have found that by neglecting the α parameter, the results concerning pIB8, pRB1 and pRB2 maintenance depend on genetic background of the host cells (MG1655, MG1655 *pcnB* and JC8679 *sbcA*). Plasmid replication and recombination can produce multimers or tangled aggregates consisting of two or more plasmid units [26, 65]. If such clusters arise, they could be segregated unevenly. After several generations, this would lead to the formation of plasmid-free cells. This reasoning is supported by experimental results, as we observed an increase of the PCN value in case of cultures after 600 generations of growth. The most dramatic effect concerning the pEC156-derivatives’ stability was observed in the case of *E*. *coli* strain deficient in the *pcnB* gene that codes for poly(A) polymerase which polyadenylates RNA molecules at the 3’ end. Such modification promotes rapid degradation of RNA molecules and produces diverse effects on bacterial metabolism [51, 66–68]. In case of ColE1-type plasmids, their copy number in *E*. *coli pcnB* is low (two copies per cell in case of pEC156-derivatives, see Table 1) due to higher stability of regulatory antisense RNA I molecules [69]. Low PCN results in the observed low stability of these replicons [35]. On the other hand, it is evident that a mutation in the *pcnB* gene not only affects plasmid replication but also impairs *E*. *coli* growth rates, suggesting that the *pcnB* gene product may have an influence on replication of chromosomal DNA [70].

In our model, we also considered importance of the post-segregational killing factor (β parameter). This parameter occasionally had to be used to improve fitting of the experimental results (Fig 1, panel G, H and I). PSK eliminates plasmid-free descendent cells. It is sufficient for the stability hypothesis that the PSK systems stabilize plasmid maintenance [71, 72] but have no effect of plasmid units segregation and do not increase the number of the plasmid-containing population [73]. Our work indicates that for the pEC156-derivatives inheritance, PSK is not as important as multimer resolution system based on the *cer*/Xer function. In conclusion, we want to stress that multimerization is a major determinant of plasmid stability. Our data indicate that even small changes in the fidelity of segregation can have serious effects on plasmid inheritance.

## Supporting information

S1 Fig Determination of plasmid multimers sizes. (PDF)

S2 Fig Principle of the method used to calculate the number of cells for a given population. (PDF)

S3 Fig Distribution of PCN in bacterial population after 0, 10, 50, 100, 150 and 200 generations. (PDF)

S4 Fig Schematic outline of plasmid stability model and its parameters. (PDF)

S5 Fig Average results of 50 simulations performed for the plasmid containing cells, employing different values of the α parameter which describes probability of the stochastic emergence of plasmid units defective in replication. (PDF)

S6 Fig Probability distribution of replication-defective plasmid units (nα) calculated for different values of the α parameter. (PDF)

S7 Fig Average results of 50 simulations performed for the plasmid containing cells, employing different values of the δ parameter, which describes uneven segregation of plasmid units between daughter cells. (PDF)

S8 Fig Theoretical prediction of plasmid copy number in bacterial populations. (PDF)

S9 Fig Simulation of distribution of cells with different plasmid copy number. (PDF)

S10 Fig Calculated distributions of pIB8 plasmid copy number in *E*. *coli* MG1655 bacterial population after 0, 250 and 600 generations. (PDF)

S1 Appendix Plasmid stability data set. (DOC)

S2 Appendix The code used to run the statistical model. (ZIP)
