# Supplementary material for: Plasmid stability analysis based on a new theoretical model employing stochastic simulations
Source: PLoS One. 2017 Aug 28;12(8):e0183512. doi: 10.1371/journal.pone.0183512 (PMC5573283; doi:10.1371/journal.pone.0183512)
Supplement: S1 Fig — (PDF) [file pone.0183512.s001.pdf]

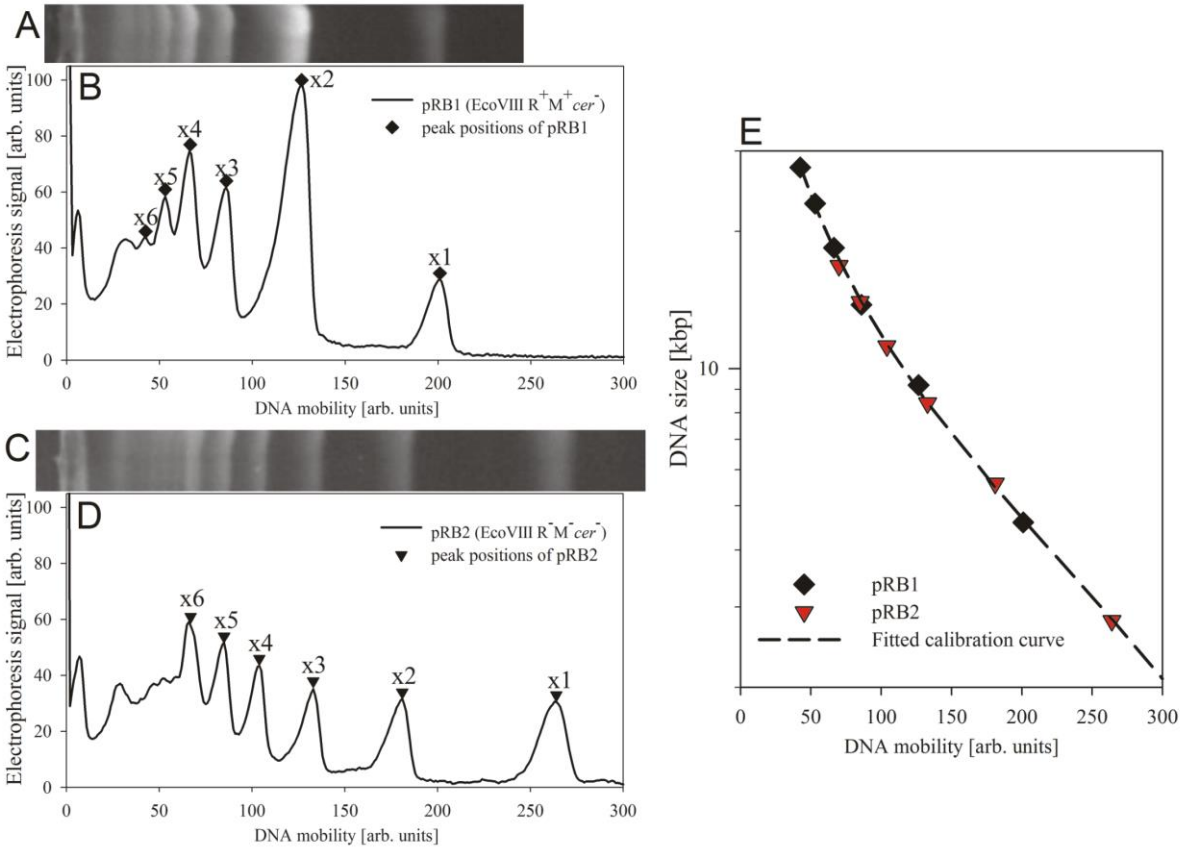


Figure S1. Determination of plasmid multimers sizes. Analyzed plasmids (pRB1 and pRB2) were isolated from hyper-recombinogenic *E. coli* JC8679 *sbcA* using an alkaline lysis method. (Panel A and C) It was assumed that plasmid multimers separated in 0.8% agarose gel represent supercoiled DNA molecules in the form of monomers, dimers, trimers, etc. (x1, x2, x3, etc.). (Panel B and D) In the next step we determined densitometrically the mobility of each plasmid multimeric form. (Panel E) These consecutive multimeric forms were clearly distinguished from one another and their peaks plotted against the theoretical size of the multimer formed a calibration curve. This curve represent nonlinear Ferguson function:

$$M(\mu)=y_0+A\cdot\ln(\mu_0/\mu) +B\cdot\ln^2(\mu_0/\mu) +C\cdot\ln^3(\mu_0/\mu)$$

where, $y_0$, A, B, C are fitting parameters
