# Supplementary material for: Plasmid stability analysis based on a new theoretical model employing stochastic simulations
Source: PLoS One. 2017 Aug 28;12(8):e0183512. doi: 10.1371/journal.pone.0183512 (PMC5573283; doi:10.1371/journal.pone.0183512)
Supplement: S2 Fig — (PDF) [file pone.0183512.s002.pdf]

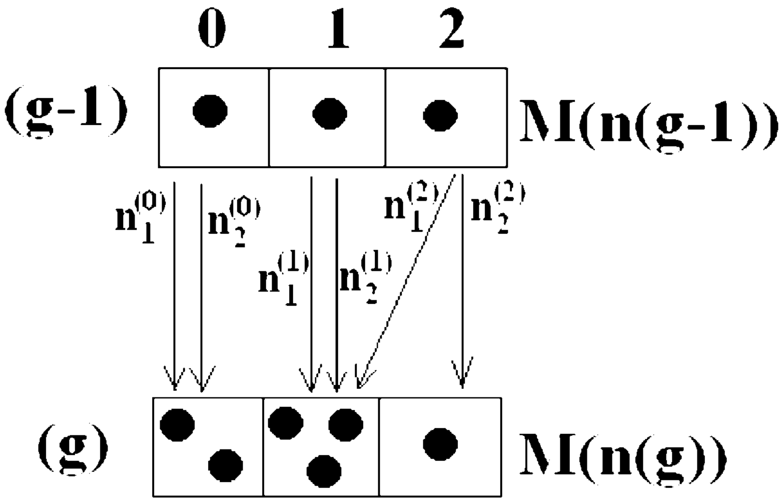

Figure S2. Principle of the method used to calculate the number of cells for a given population. Initially, at (g-1) generation there are three cells, with n=2, n=1, and n=0, where n indicates plasmid copy number. In the next generation (g) due to random processes, there will be 6 newborn cells, one with n=2, three cells with n=1 and two cells with n=0
