# Supplementary material for: Plasmid stability analysis based on a new theoretical model employing stochastic simulations
Source: PLoS One. 2017 Aug 28;12(8):e0183512. doi: 10.1371/journal.pone.0183512 (PMC5573283; doi:10.1371/journal.pone.0183512)
Supplement: S3 Fig — (PDF) [file pone.0183512.s003.pdf]

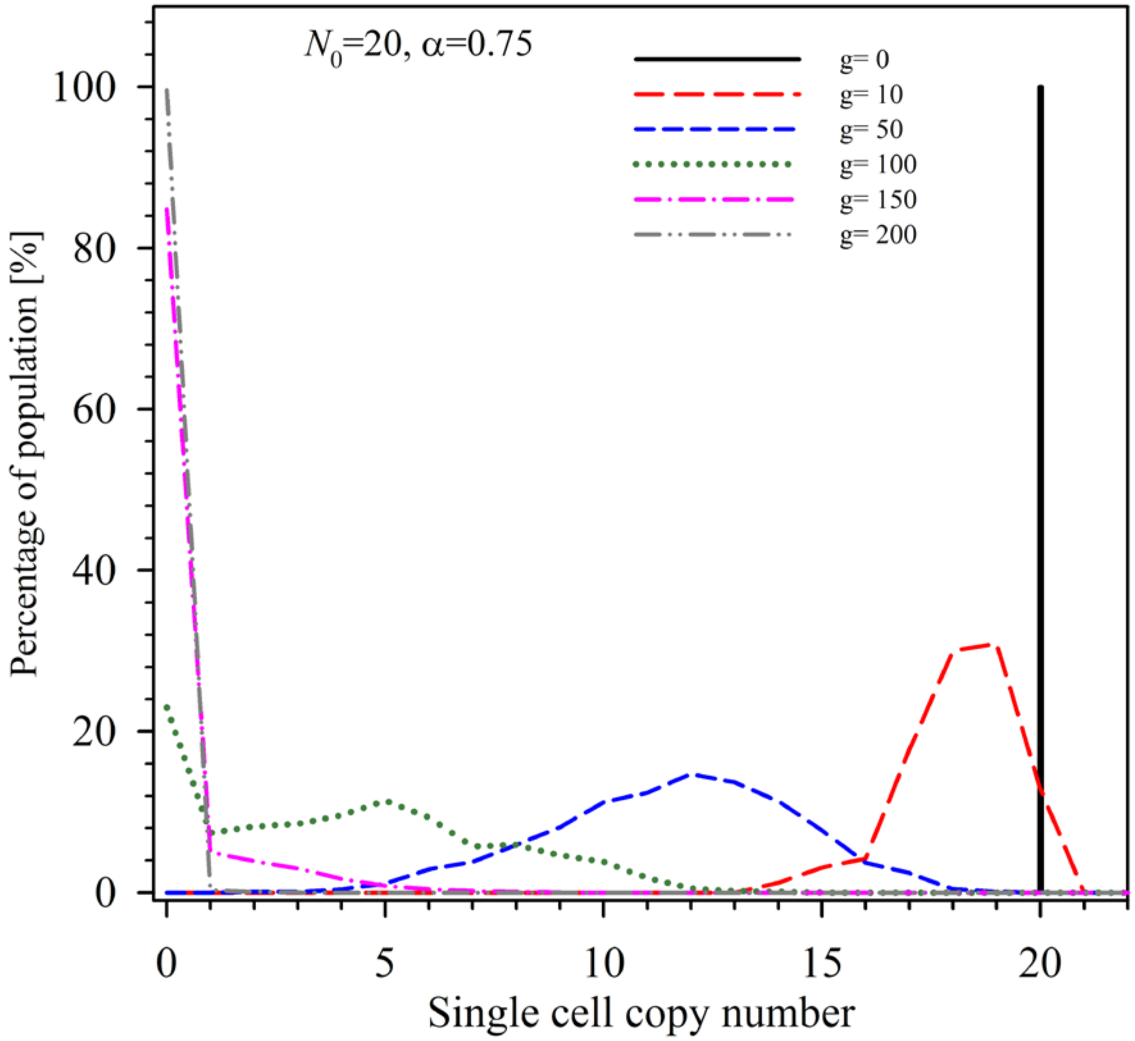

Figure S3. Distribution of PCN in bacterial population after 0, 10, 50, 100, 150 and 200 generations. Initial PCN distribution is derived from equation (9), and N_0=20 and α=0.75 values were adopted.
