# Supplementary material for: Plasmid stability analysis based on a new theoretical model employing stochastic simulations
Source: PLoS One. 2017 Aug 28;12(8):e0183512. doi: 10.1371/journal.pone.0183512 (PMC5573283; doi:10.1371/journal.pone.0183512)
Supplement: S4 Fig — (PDF) [file pone.0183512.s004.pdf]

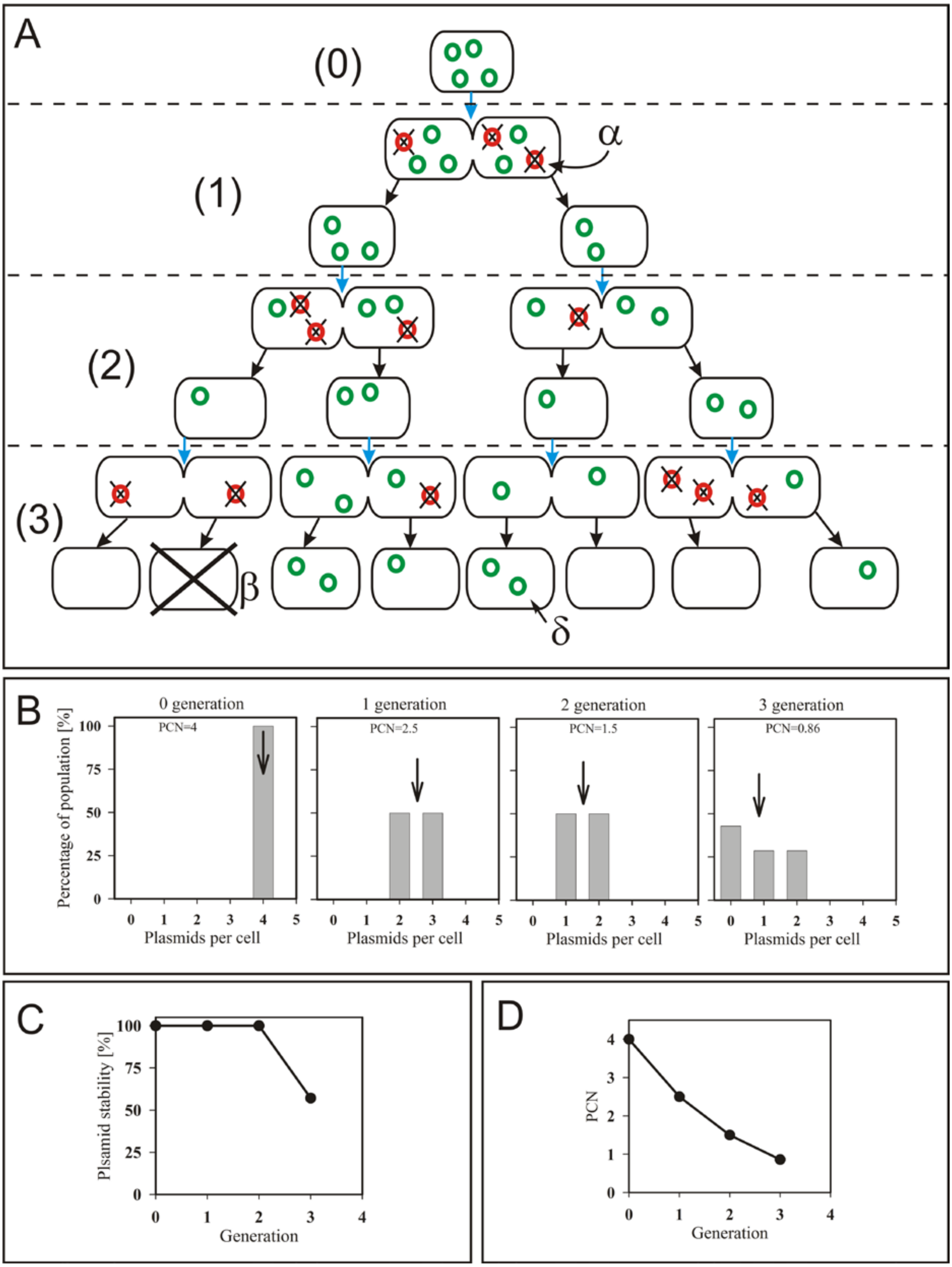

Figure S4. Schematic outline of plasmid stability model and its parameters. (Panel A) Plasmid distribution after three consecutive cell divisions. Green circles indicate functional plasmid units, red crossed circles indicate plasmid units that are defective in replication. Probability of

their emergence is described by the α parameter. Post-segregational death rate of cells that lost the plasmid is defined by the β parameter. At each generation, the top row represents plasmid content after cell division, while the bottom row shows only fully functional plasmid units (green circles). (Panel B) Relative distribution of the plasmid copy number resulting from three consecutive cell divisions. Arrows indicate the mean plasmid copy number of the final distribution. (Panel C) Plasmid stability derived from the proposed model, $S(g)$ (Eq. 11). (Panel D) The average number of plasmid units per generation, $P(n,g)$ (Eq. 10).
