# Supplementary material for: Plasmid stability analysis based on a new theoretical model employing stochastic simulations
Source: PLoS One. 2017 Aug 28;12(8):e0183512. doi: 10.1371/journal.pone.0183512 (PMC5573283; doi:10.1371/journal.pone.0183512)
Supplement: S5 Fig — (PDF) [file pone.0183512.s005.pdf]

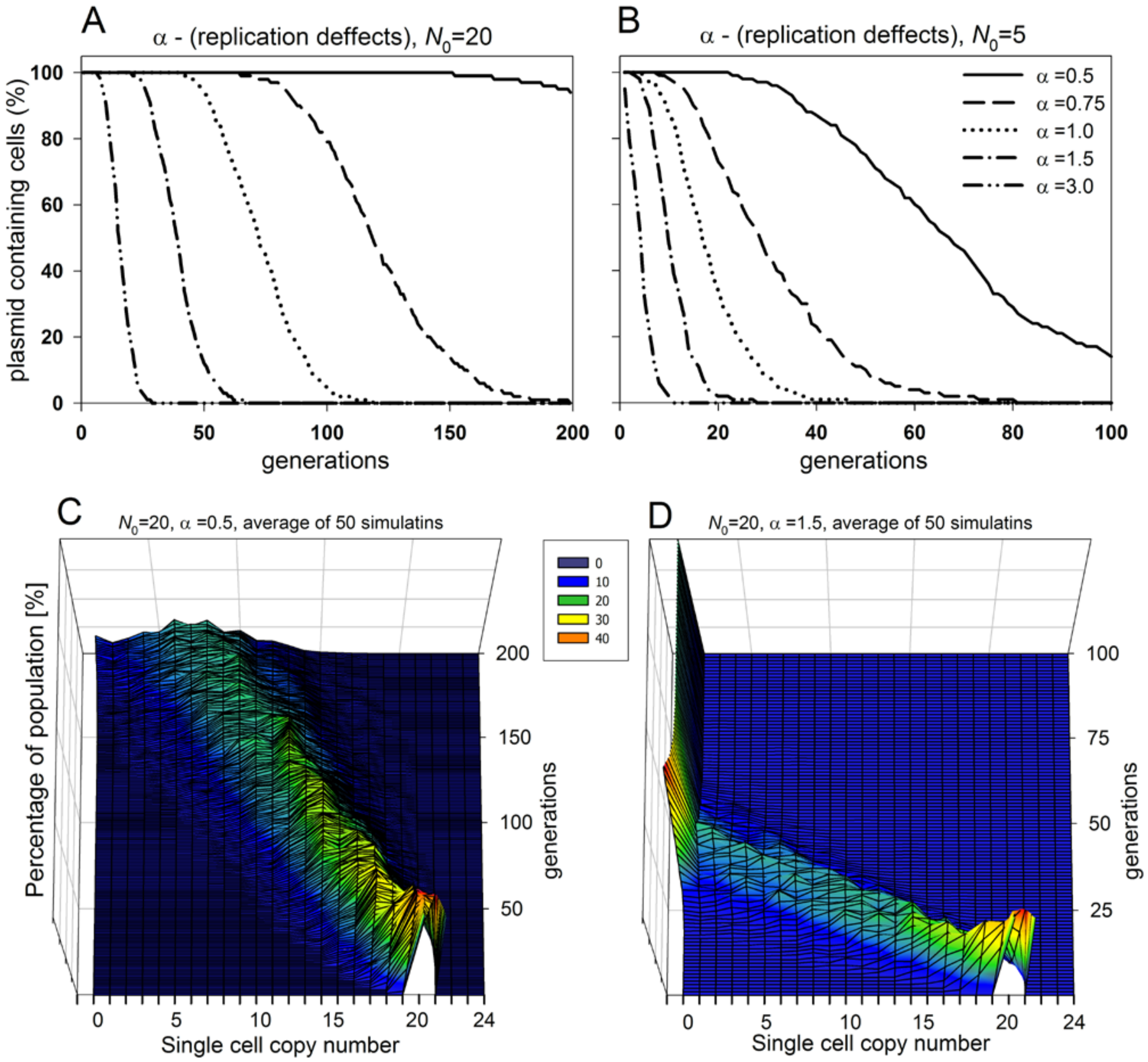

Figure S5. Average results of 50 simulations performed for the plasmid containing cells, employing different values of the α parameter which describes probability of the stochastic emergence of plasmid units defective in replication. The initial parameters are: uniform distribution described by Eq. 9 with (Panel A) N_0=20 and (Panel B) N_0=5 (δ=0). The 3-D plots show distribution of a single cell copy number in a bacterial population for the following parameters: N_0=20 and α=0.5 (C) and α=1.5 (D).
