# Supplementary material for: Plasmid stability analysis based on a new theoretical model employing stochastic simulations
Source: PLoS One. 2017 Aug 28;12(8):e0183512. doi: 10.1371/journal.pone.0183512 (PMC5573283; doi:10.1371/journal.pone.0183512)
Supplement: S6 Fig — (PDF) [file pone.0183512.s006.pdf]

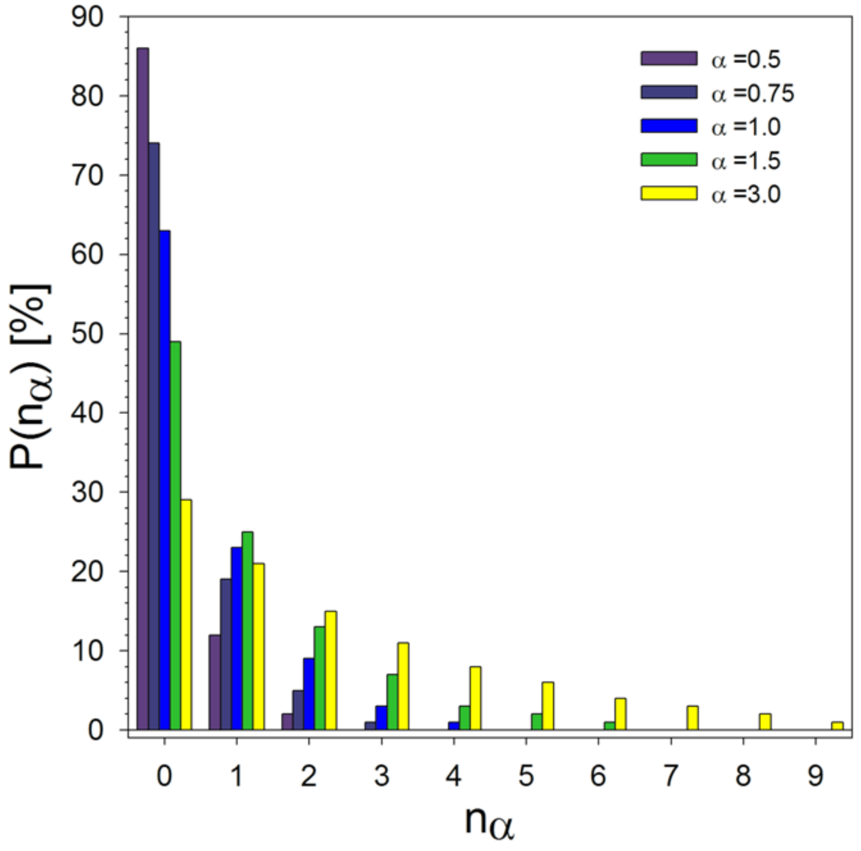

Figure S6. Probability distribution of replication-defective plasmid units (n_α) calculated for different values of the α parameter.
