# Supplementary material for: Plasmid stability analysis based on a new theoretical model employing stochastic simulations
Source: PLoS One. 2017 Aug 28;12(8):e0183512. doi: 10.1371/journal.pone.0183512 (PMC5573283; doi:10.1371/journal.pone.0183512)
Supplement: S7 Fig — (PDF) [file pone.0183512.s007.pdf]

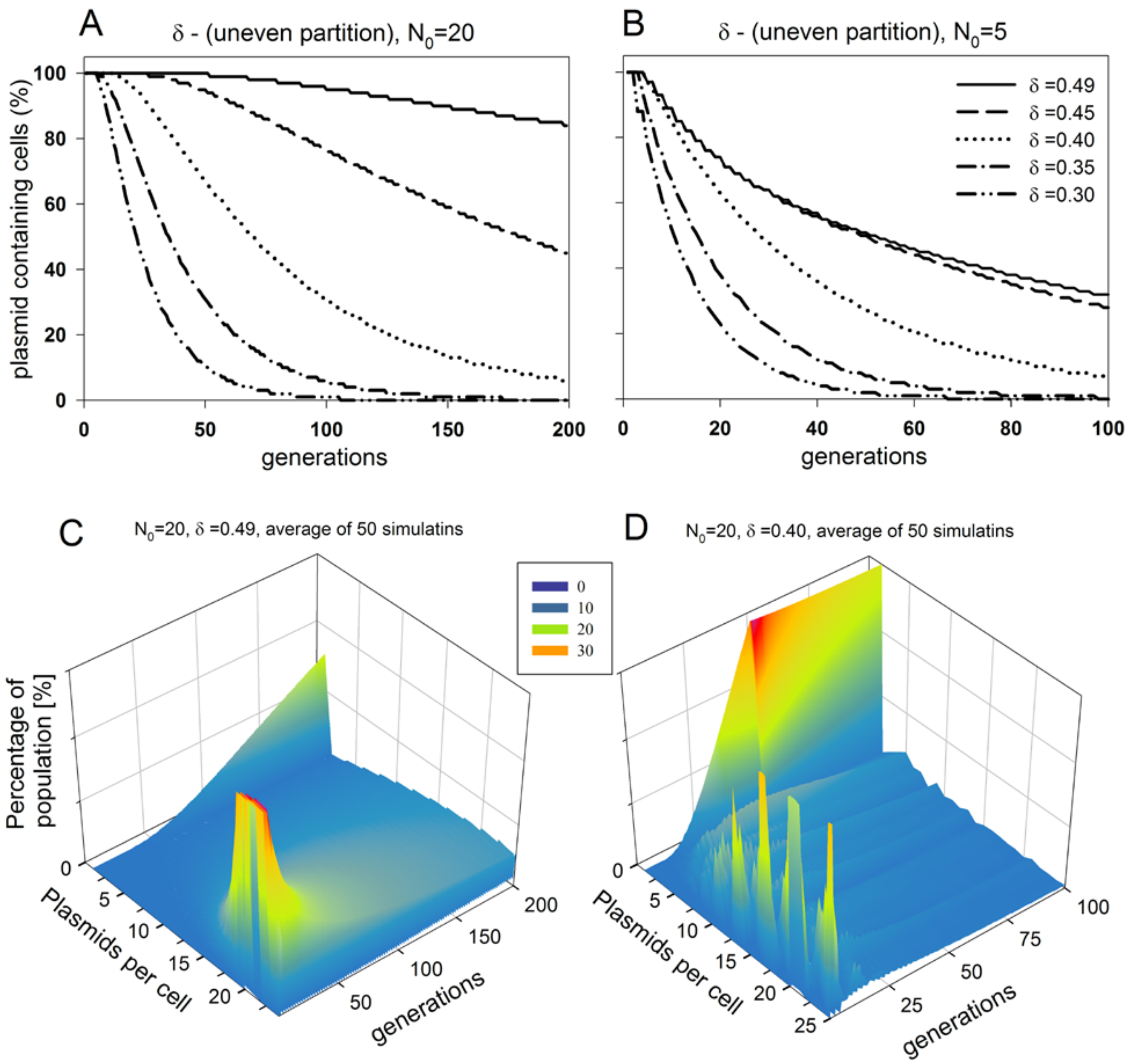

Figure S7. Average results of 50 simulations performed for the plasmid containing cells, employing different values of the δ parameter, which describes uneven segregation of plasmid units between daughter cells. The initial parameters are: uniform distribution described by Eq. 9 with N_0=20 (Panel A) and N_0=5 (Panel B) (α=0). The 3-D plots show distribution of a single cell copy number in a bacterial population for the following parameters: N_0=20 and δ=0.49 (C) and δ=0.40 (D).
