# Supplementary material for: Plasmid stability analysis based on a new theoretical model employing stochastic simulations
Source: PLoS One. 2017 Aug 28;12(8):e0183512. doi: 10.1371/journal.pone.0183512 (PMC5573283; doi:10.1371/journal.pone.0183512)
Supplement: S8 Fig — (PDF) [file pone.0183512.s008.pdf]

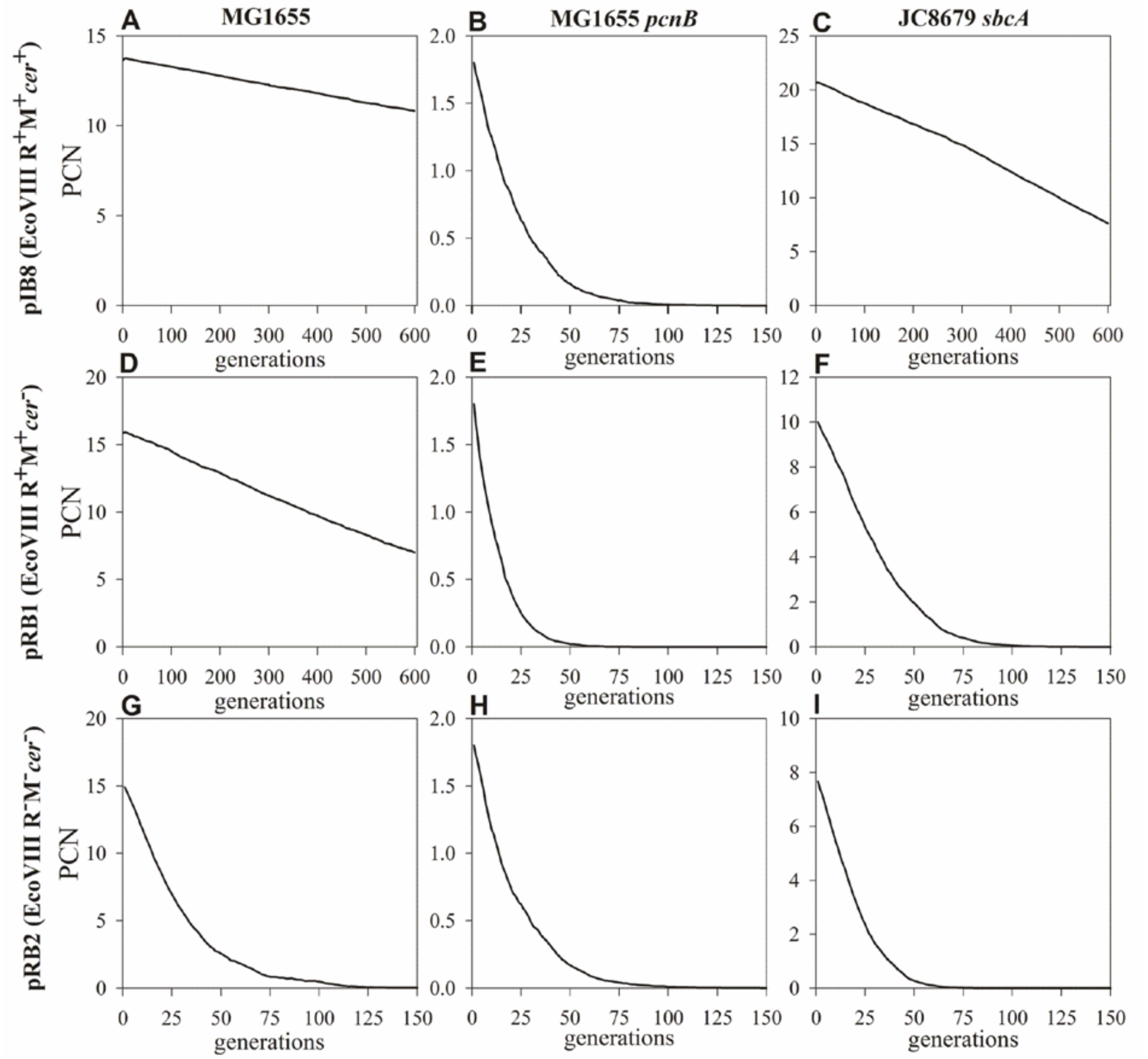

Figure S8. Theoretical prediction of plasmid copy number in bacterial populations. As hosts *E. coli* MG1655 (wild type), MG1655 *pcnB* and hyper-recombinogenic JC8679 *sbcA* were used.
