# Supplementary material for: Plasmid stability analysis based on a new theoretical model employing stochastic simulations
Source: PLoS One. 2017 Aug 28;12(8):e0183512. doi: 10.1371/journal.pone.0183512 (PMC5573283; doi:10.1371/journal.pone.0183512)
Supplement: S10 Fig — (PDF) [file pone.0183512.s010.pdf]

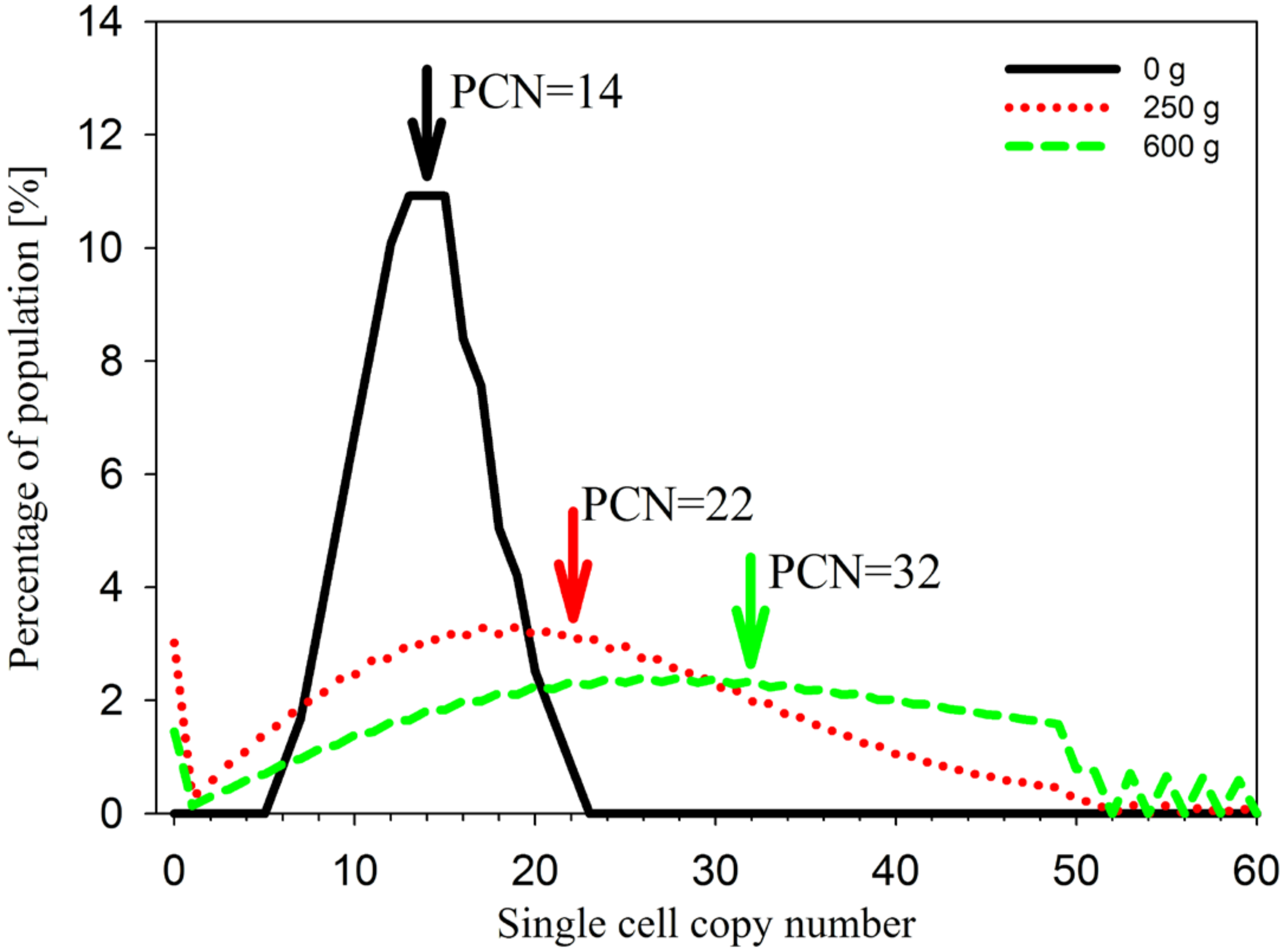

Figure S10. Calculated distributions of pIB8 plasmid copy number in *E. coli* MG1655 bacterial population after 0, 250 and 600 generations. Initial parameters are the same as in Fig. 2A, N_0=14 and δ=0.49 values were adopted. Vertical arrows indicate an average of plasmid copy numbers determined for each distribution.
