# Supplementary material for: Plasmid stability analysis based on a new theoretical model employing stochastic simulations
Source: PLoS One. 2017 Aug 28;12(8):e0183512. doi: 10.1371/journal.pone.0183512 (PMC5573283; doi:10.1371/journal.pone.0183512)
Supplement: S1 Appendix — (DOC) [file pone.0183512.s011.doc]

**Appendix S1**

**Plasmid stability data set**

First row indicates number of generations. Roman numerals indicate experiments which were run in parallel.

*E. coli* MG1655[pIB8] (this work)

I II III Mean Standard deviation

25 100% 100% 100% 100 0

50 100% 100% 100% 100 0

75 93% 100% 100% 97.66666667 4.041451884

100 95% 100% 100% 98.33333333 2.886751346

125 100% 100% 100% 100 0

150 88% 100% 95% 94.33333333 6.027713773

175 90% 100% 92% 94 5.291502622

200 100% 90% 94% 94.66666667 5.033222957

225 95% 100% 94% 96.33333333 3.214550254

250 100% 97% 97% 98 1.732050808

275 100% 95% 100% 98.33333333 2.886751346

300 100% 100% 99% 99.66666667 0.577350269

325 100% 100% 98% 99.33333333 1.154700538

350 100% 100% 94% 98 3.464101615

375 96% 98% 95% 96.33333333 1.527525232

400 99% 100% 99% 99.33333333 0.577350269

425 100% 98% 100% 99.33333333 1.154700538

450 95% 87% 97% 93 5.291502622

500 100% 95% 98% 97.66666667 2.516611478

550 97% 97% 94% 96 1.732050808

600 100% 98% 94% 97.33333333 3.055050463

*E. coli* MG1655[pRB1] (this work)

I II III Mean Standard deviation

25 100% 100% 100% 100 0

50 100% 100% 100% 100 0

75 98% 97% 100% 98.33333333 1.527525232

100 100% 84% 100% 94.66666667 9.237604307

125 94% 100% 100% 98 3.464101615

150 82% 100% 100% 94 10.39230485

175 96% 100% 100% 98.66666667 2.309401077

200 97% 100% 93% 96.66666667 3.511884584

225 96% 95% 94% 95 1

250 100% 97% 99% 98.66666667 1.527525232

275 100% 95% 97% 97.33333333 2.516611478

300 100% 100% 97% 99 1.732050808

325 100% 100% 98% 99.33333333 1.154700538

350 100% 100% 81% 93.66666667 10.96965511

375 96% 98% 80% 91.33333333 9.865765725

400 99% 100% 93% 97.33333333 3.785938897

425 100% 98% 94% 97.33333333 3.055050463

450 95% 87% 90% 90.66666667 4.041451884

500 100% 95% 96% 97 2.645751311

550 97% 97% 99% 97.66666667 1.154700538

600 87% 98% 80% 88.33333333 9.073771726

*E. coli* MG1655[pRB2] (this work)

I II III Mean Standard deviation

0 100% 100% 100% 100 0

25 98% 100% 79% 92.33333333 12

50 65% 82% 34% 60.33333333 24

75 35% 49% 16% 33.33333333 17

100 23% 35% 23% 27 7

*E. coli* MG1655 *pcnB*[pIB8] (data presented previously [1])

I II Mean Standard deviation

0 100% 100% 100 0

25 36% 40% 38 3

50 8.5% 10% 9.25 1

75 6% 5% 5.5 1

100 1% 0 0.5 1

*E coli* MG1655 *pcnB*[pRB1] (data presented previously [1])

I II Mean Standard deviation

0 100% 100% 100 0

25 10% 19% 14.5 6

50 2% 5% 3.5 2

75 0 0 0 0

100 0 0 0 0

*E coli* MG1655 *pcnB*[pRB2] (this work)

I II Mean Standard deviation

0 100% 100% 100 0

25 17% 35% 26 13

50 20% 9% 14.5 8

75 6% 2% 4 3

100 1% 0 0.5 1

*E. coli* JC8679 *sbcA*[pIB8] (this work)

I II III IV Mean Standard deviation

25 100% 100% 100% 100% 100 0

50 100% 100% 100% 99% 99.75 0.5

75 100% 98% 100% 98% 99 1.154700538

100 100% 100% 100% 99% 99.75 0.5

125 100% 100% 100% 100% 100 0

150 100% 100% 100% 98% 99.5 1

175 96% 98% 94% 100% 97 2.581988897

200 100% 100% 95% 96% 97.75 2.62995564

225 98% 100% 94% 93% 96.25 3.304037934

250 100% 98% 99% 93% 97.5 3.109126351

275 100% 95% 97% 100% 98 2.449489743

300 100% 100% 97% 95% 98 2.449489743

325 100% 96% 98% 94% 97 2.581988897

350 100% 88% 81% 100% 92.25 9.394147114

375 96% 98% 80% 93% 91.75 8.098353742

400 99% 100% 93% 100% 98 3.366501646

425 100% 84% 94% 97% 93.75 6.946221995

450 95% 87% 90% 96% 92 4.242640687

500 99% 96% 96% 96% 96.75 1.5

550 97% 85% 99% 95% 94 6.218252702

600 95% 98% 90% 85% 92 5.715476066

*E. coli* JC8679 *sbcA*[pRB2] (data presented previously [1])

I II III IV Mean Standard deviation

0 100% 100% 100% 100% 100 0

25 55.5% 49% 75% 42% 55.375 14

50 15.5% 22% 15% 8% 15.125 6

75 1% 5.08% 1% 1% 2.02 2

100 0 3.51% 0 0 0.8775 2

*E. coli* JC8679 *sbcA* [pRB1] (data presented previously [1])

I II III Mean Standard deviation

0 100% 100% 100% 100 0

25 50% 54.5% 45% 50 5

50 47% 50.75% 43% 47 4

75 21% 33.26% 12% 22 11

100 12% 23.41% 4% 13 10

1. Werbowy O, Boratynski R, Dekowska A, Kaczorowski T. Genetic analysis of maintenance of pEC156, a naturally occurring *Escherichia coli* plasmid that carries genes of the EcoVIII restriction-modification system. Plasmid. 2015;77:39-50. Epub 2014/12/08. doi: 10.1016/j.plasmid.2014.12.002. PubMed PMID: 25500017.
