# Supplementary figures and images for: Plasmid stability analysis based on a new theoretical model employing stochastic simulations
Source: PLoS One. 2017 Aug 28;12(8):e0183512. doi: 10.1371/journal.pone.0183512 (PMC5573283; doi:10.1371/journal.pone.0183512)

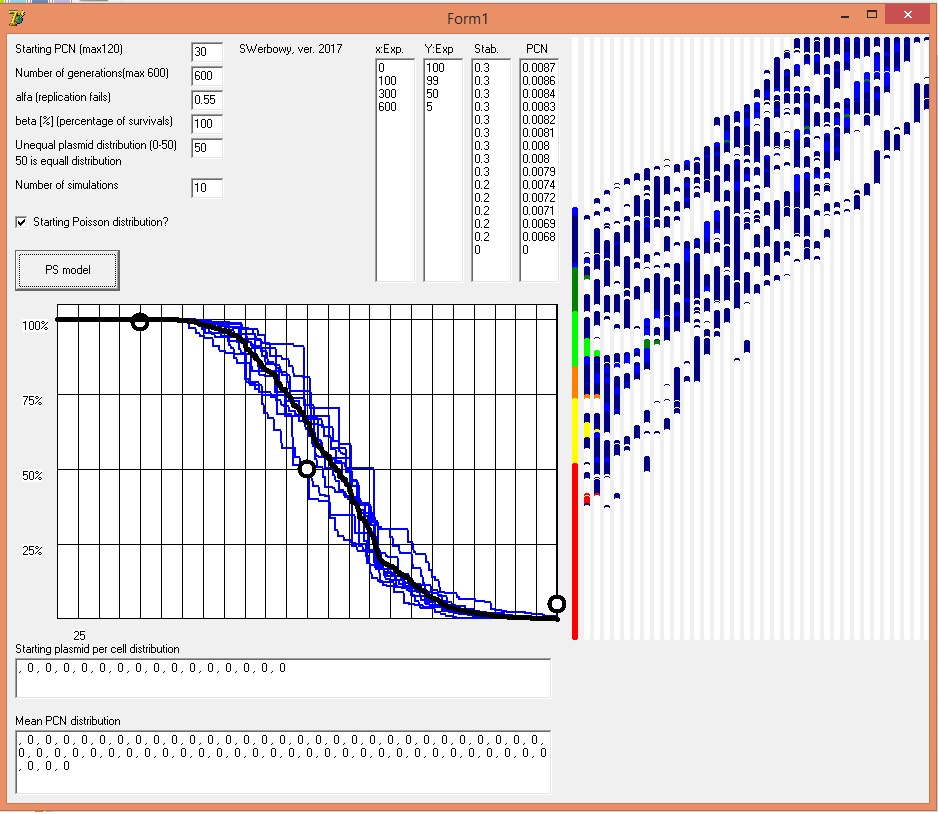

Supplement: S2 Appendix — (ZIP) [file pone.0183512.s012.zip › Program-PrintScreen.jpg]
